# Spatiotemporal profiling of functional network overlapping modules in Alzheimer’s disease

**DOI:** 10.1162/NETN.a.516

**Published:** 2026-01-28

**Authors:** Yue Gu, Ying Lin, Liangfang Li, Junji Ma, Sihan Wei, Zhengjia Dai, for the Alzheimer’s Disease Neuroimaging Initiative

**Affiliations:** Department of Psychology, Sun Yat-sen University, Guangzhou, China

**Keywords:** Overlapping modular organization, Multilayer dynamic networks, Temporal variability, Overlapping node, Alzheimer’s disease

## Abstract

Alzheimer’s disease (AD) is characterized by progressive neural network degradation. In brain functional networks, overlapping module structures provide more accurate representations of brain function than nonoverlapping structures. Since the involvement of overlapping nodes in multiple modules can vary over time, investigating dynamic functional changes in the brain may provide deeper insights into the structural characteristics of these overlapping modules. However, the spatiotemporal dynamics of overlapping modular brain organization remain unclear. We employed resting-state fMRI to explore the overlapping modular organization and dynamic multilayer modules in 64 AD (Age_mean_ = 74.04) and 61 healthy controls (HC, Age_mean_ = 74.86) from the Alzheimer’s Disease Neuroimaging Initiative. Compared with HC, AD exhibited increased overlapping modules and decreased modularity, with altered nodal overlapping probability, particularly in the superior frontal cortex and hippocampus. Higher nodal overlapping probability correlated with greater flexibility and was associated with larger amyloid deposits. Lasso regression analysis further revealed strong correlations between overlapping nodal characteristics and cognitive performance. Our findings suggest that overlapping nodes are critical components in AD, demonstrating high amyloid deposition, significant functional flexibility, and strong associations to cognitive behavior. These alterations may enhance the understanding of AD pathology and contribute to the development of biomarkers for improved diagnosis and therapeutic strategies.

## Author Summary

Alzheimer’s disease (AD) is characterized by progressive neural network degradation, yet the spatiotemporal dynamics of overlapping modular brain organization remain poorly understood. Through investigation of overlapping modular organization and dynamic multilayer modules in AD, we demonstrated that AD is associated with increased numbers of overlapping modules and decreased modularity, accompanied by altered nodal overlapping probability, particularly in the superior frontal cortex and hippocampus. Notably, higher nodal overlapping probability was correlated with increased temporal variability, greater functional flexibility, and larger amyloid deposits. Lasso regression analysis revealed strong associations between overlapping nodal characteristics and cognitive performance. Our findings underscore the critical role of overlapping nodes in AD pathophysiology, providing insights into disease mechanisms and potential avenues for biomarker development and therapeutic interventions.

## INTRODUCTION

Alzheimer’s disease (AD), the most prevalent neurodegenerative dementia, is characterized by progressive cognitive decline and behavioral changes (Adaszewski et al., 2013). Its increasing prevalence among aging populations places substantial strain on healthcare systems and exacerbates social challenges, including stigma and isolation (Cuijpers & van Lente, 2015). Enhanced understanding of AD mechanisms can facilitate early diagnosis and treatment, potentially improving quality of life for affected individuals.

Since the advent of functional magnetic resonance imaging (fMRI) in human brain research, this technique has revolutionized the study of neural architecture (Biswal & Uddin, 2025). Compared with anatomical networks, functional networks demonstrate greater dynamism and flexibility (Bassett & Sporns, 2017). fMRI also demonstrates effectiveness in capturing brain-behavior relationships, as highlighted by meta-analyses (Biswal & Uddin, 2025; Crossley et al., 2013). Importantly, studies based on resting-state fMRI (rs-fMRI) indicate that changes in brain function may occur before the emergence of obvious clinical symptoms or structural damage, highlighting that fMRI can indirectly measure early brain dysfunction in AD (Dennis & Thompson, 2014). As a complex system, the brain network continuously processes and transmits information between spatially distributed regions (Bullmore & Sporns, 2009). Modules, one of the most essential topological features of brain networks, are subnetworks that are tightly connected internally but loosely connected with other modules (Sporns, 2018). Researchers have employed different modalities and computational methods to investigate modular organization and information communication in AD (Dai & He, 2014). For example, a decreased number of larger modules and reduced modularity have been found in AD compared with healthy controls (HCs) (Ng et al., 2021). Additionally, in the early stages of AD, module-related characteristics have been shown to be informative in discovering disorder-related changes (Rathore et al., 2017).

However, previous studies of modules have typically assumed that each brain node belongs to a single functional module, overlooking the potential spatial overlap between modules. Indeed, the structures of real-world networks often exhibit overlapping properties, where regions can participate in multiple modules. Increasing evidence indicates that overlapping organization offers a more realistic representation of brain function compared with nonoverlapping modules because brain regions can participate in multiple cognitive tasks (Braun et al., 2015; Faskowitz et al., 2020). By considering overlapping connectivity, researchers can better identify patterns related to cognitive function and dysfunction, particularly in conditions such as AD. Currently, there is limited research on overlapping modular structures in AD. One study revealed that AD patients exhibit significant alterations in the overlapping modular structure of functional networks, particularly in the frontoparietal and basal ganglia networks (Han et al., 2022). For patients with AD, overlapping modular structures enhance adaptability and the capacity to reorganize in response to interventions such as repetitive transcranial magnetic stimulation more than nonoverlapping structures (Yao et al., 2025). Overlapping nodes, which participate in multiple modules, exhibit functional multimodality and structural hub properties, acting as critical nodes for cross-module information integration. Their dysfunction may be associated with hub vulnerability and impaired network integration in cognitive disorders (Jo et al., 2021), but the relationship between overlapping nodes and cognitive functions enabling flexibility is not well understood. Furthermore, the pathophysiology of overlapping modules in AD remains unclear.

Dynamic functional connectivity facilitates the observation of details that are averaged out in static functional connectivity and has been identified as a potentially sensitive biomarker for neurodegenerative disorders (Biswal & Uddin, 2025; Gu et al., 2020). As the involvement of overlapping nodes in multiple modules can change over time, dynamic functional connectivity may enhance our understanding of overlapping modular structures. The dynamic multilayer modular approach, calculated through dynamic functional connectivity, involves structures within multilayer networks that capture interactions across different layers over time, providing insights into how brain regions communicate in various contexts (Liu et al., 2020). Previous studies have suggested using the multilayer framework to detect AD and have found that dynamic multilayer modular measures exhibit strong associations with cognition as well as with amyloid and tau pathology (Avelar-Pereira et al., 2023; Canal-Garcia et al., 2024). Similarly, this approach has helped identify potential biomarkers for other disorders, such as schizophrenia and bipolar disorder (Hu et al., 2025; Lai et al., 2024). It is necessary to determine whether dynamic multilayer modules maintain biomarker sensitivity and to elucidate the impact of their dynamic characteristics on overlapping modular structure and temporal transitions of brain region affiliations in AD.

**Figure 1.**
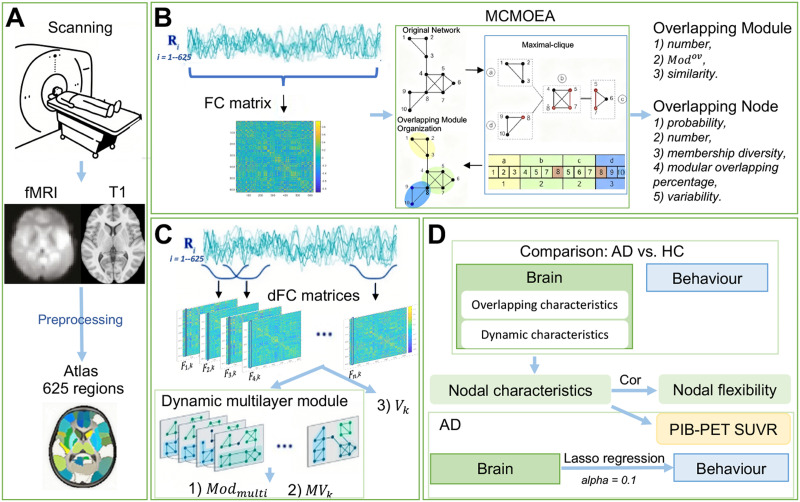
Overview of the methodological pipeline. Abbreviations include dFC (dynamic functional connectivity), Cor (correlation), MCMOEA (maximal clique-based multiobjective evolutionary algorithm), *Mod*^*ov*^ (overlapping modularity score), *Mod*_*multi*_ (dynamic modularity), *MV*_*k*_ (dynamic modular variability), *V*_*k*_ (nodal temporal variability), and SUVR (standardized uptake value ratio).

To address these issues, we investigated the overlapping modular organization of the human brain functional network using rs-fMRI, exploring its interactions with dynamic multilayer modules in 64 AD and 61 HC participants. To identify individual overlapping modular structures, we employed the maximal-clique-based multi-objective evolutionary algorithm (Lin et al., 2018; Wen et al., 2017). Based on the detected overlapping modules, we calculated characteristics of overlapping modules and nodes to identify abnormal changes in AD. The organization of overlapping modules is closely linked to variations in brain activity, whereby dynamic multilayer modules may enhance our understanding of these overlapping configurations. Notably, overlapping nodes are likely to correspond to those exhibiting high temporal variability, reflecting their integral role in the brain’s adaptive processes. Consequently, we constructed multilayer modules. Consequently, we organized multilayer modules (Liu et al., 2020), and calculated temporal variability of regional functional connectivity across the entire time series (Gu et al., 2020; Zhang et al., 2016). We hypothesize that overlapping nodes and nodes exhibiting high variability are critical components of the brain’s network architecture, characterized by significant functional flexibility. In the context of AD, these nodes may be particularly vulnerable to pathological insults, leading to increased susceptibility and subsequent cognitive decline. We calculated the relationships between functional characteristics—specifically nodal overlapping probability and dynamic modular variability—and functional flexibility (Yeo et al., 2015) as well as amyloid deposition (La Joie et al., 2020). Finally, we examined how module-related characteristics were associated with cognitive performance in AD. Figure 1 summarizes the overall methodological pipeline.

## RESULTS

### Static Overlapping Module Structures

**Figure 2.**
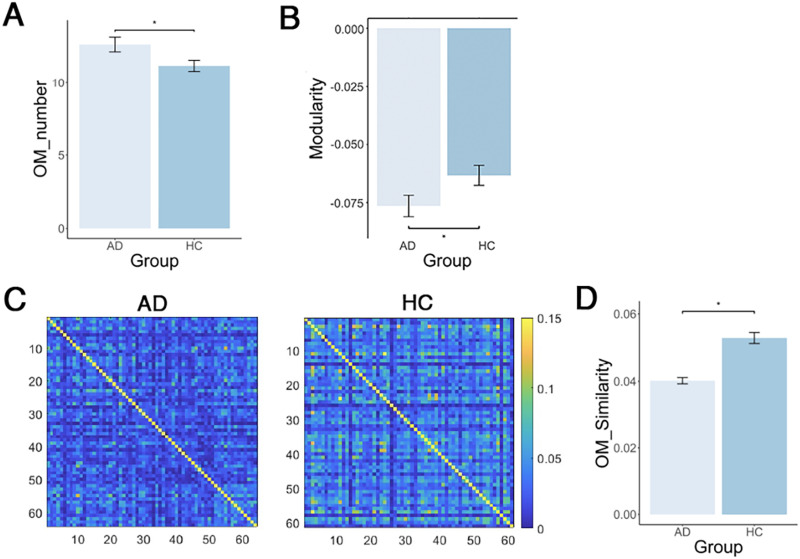
Differences in overlapping modular characteristics between AD and HC. (A) The differences in the number of overlapping modules between AD and HC. (B) Comparison of overlapping modularity between AD and HC. (C) The similarity of individual overlapping modular structures within AD and HC groups. The color bar represents the similarity values, with higher values indicating greater similarity in overlapping modular structures. (D) The between-group differences in this similarity. The asterisks indicate significant between-group differences (*p* < 0.05).

Based on the overlapping modular architecture obtained, we identified significant between-group differences in all three overlapping module (OM) characteristics. Specifically, compared with HC, AD demonstrated significantly higher values in the number of overlapping modules (*p* = 0.015, *t* = 2.429, Cohen’s *d* (*d*) = 0.432; Figure 2A), and lower value in overlapping modularity (*p* = 0.027, *t* = −2.222, *d* = −0.398; Figure 2B). With regard to the similarity among the individual overlapping modular structures (Figure 2C), we found a significant decrease in modular similarity for AD compared with HC (*p* = 0.002, *t* = −17.534, *d* = −0.574; Figure 2D).

**Figure 3.**
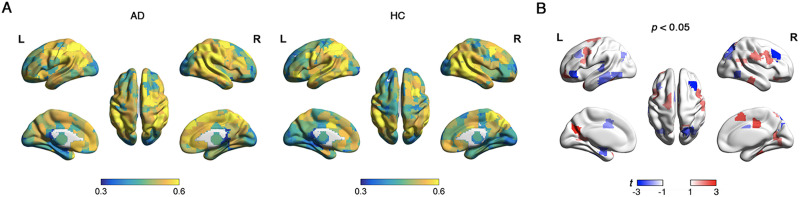
The nodal overlapping probability in AD and HC. (A) Spatial distribution of overlapping probabilities in AD and HC groups. The color bar represents the values of overlapping probabilities, with higher values indicating that nodes have a greater probability of overlap. (B) Between-group differences based on the *t* test. Red coloring indicates higher *t* values in AD compared with HC, whereas blue coloring indicates lower *t* values in AD relative to HC.

**Figure 4.**
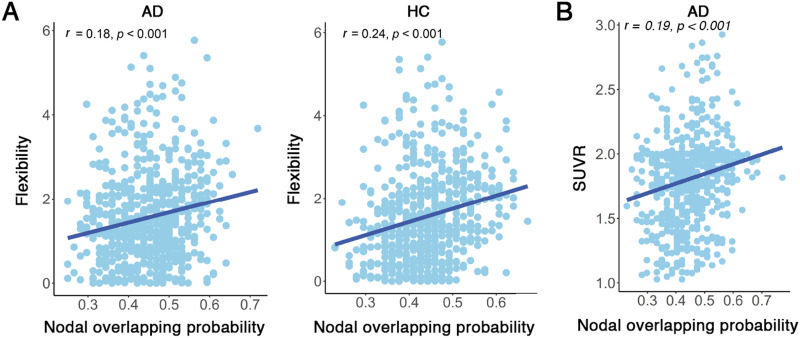
(A) The correlation between nodal overlapping probability and functional flexibility in both AD and HC groups. (B) Correlation between nodal overlapping probability and SUVR indicating amyloid deposits in the brain of AD patients. Each point represents a brain node.

There were no significant differences between AD and HC groups in overlapping node characteristics, except for nodal overlapping probability. In both AD and HC groups, regions exhibiting the highest overlapping probabilities were predominantly located in the right middle temporal cortex and left superior parietal lobe, whereas regions with the lowest overlapping probabilities were primarily localized in the occipital lobe and left hippocampus (Figure 3A). Significant between-group differences were identified in 42 nodes regarding overlapping probability (*p* < 0.05, 1.868 < |*t*| < 3.030, 0.335 < |*d*| < 0.544, non-false discovery rate (FDR) correction; Figure 3B). Among these, 24 nodes exhibited higher overlapping probability in AD than in HC, with most located in the inferior and superior frontal cortex, superior temporal cortex, superior parietal lobe, fusiform gyrus, cuneus, middle cingulate cortex, and hippocampus. Eighteen nodes demonstrated lower overlapping probabilities in AD than in HC, which were distributed in the middle and inferior frontal cortex, inferior temporal cortex, middle occipital cortex, and hippocampus. Nodal overlapping probability was positively correlated with functional flexibility in both AD (*r* = 0.184, *p* < 0.001) and HC (*r* = 0.241, *p* < 0.001) groups (Figure 4A). These results suggest that overlapping nodes are associated with greater functional flexibility. Additionally, the SUVR serves as a critical metric for assessing amyloid deposition in the brain of AD. In AD patients, nodal overlapping probability demonstrated a positive correlation with SUVR (*r* = 0.189, *p* < 0.001; Figure 4B), suggesting that overlapping nodes are associated with higher concentrations of amyloid deposits in AD.

### Dynamic Module Structures

**Figure 5.**
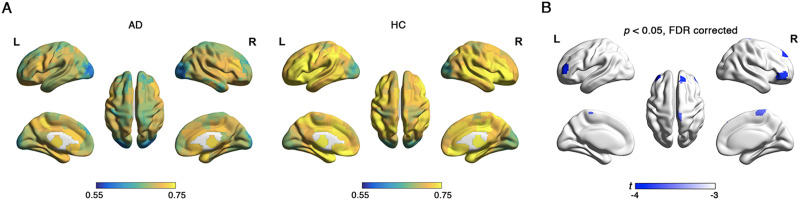
The temporal variability in module affiliations. (A) Spatial distribution of the temporal variability in module affiliations across all time windows in AD and HC groups. The color bar represents the values of temporal variability in module affiliations, with higher values indicating greater variability in the structure of module. (B) Group comparison showing changes in the temporal variability of AD compared with HC.

**Figure 6.**
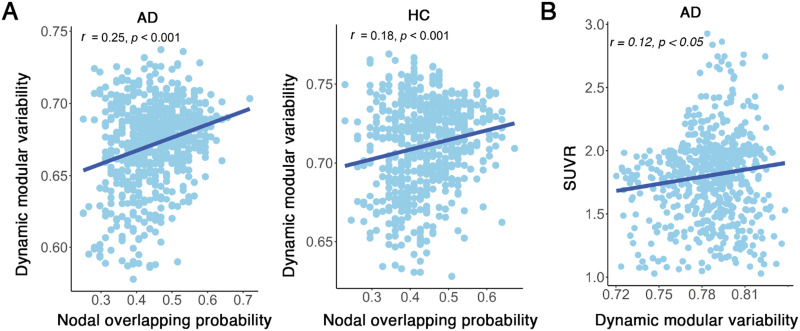
(A) The correlation between the temporal modular variability and the nodal overlapping probability in AD and HC groups. (B) Correlation between temporal modular variability and SUVR indicating amyloid deposits in the brain of AD patients. Each point represents a brain node.

For each participant, we observed that network nodes dynamically switched their modular affiliations across time windows, indicating that the modular structure of the brain network was temporally variable. Comparing with HC, AD demonstrated significantly larger dynamic modularity (*p* = 0.005, *t* = 2.782, *d* = 0.495; Supporting Information Figure S1). The temporal variability in module affiliations across all time windows was spatially heterogeneous, with high variability predominantly located in the frontal and temporal cortices (Figure 5A). Specifically, AD exhibited significantly decreased temporal modular variability in the frontal cortex (*p* < 0.001, −4.450 < *t* < −3.346, −0.803 < *d* < −0.592; Figure 5B). Notably, the temporal modular variability that captured dynamic modular reorganization was positively correlated with nodal overlapping probability in the static modular structure (AD: *r* = 0.253, HC: *r* = 0.181, *p* < 0.001; Figure 6A). These findings indicate that nodes exhibiting higher dynamic modular variability over time tend to be overlapping nodes. Additionally, temporal modular variability demonstrated a positive correlation with SUVR in AD (*r* = 0.124, *p* = 0.001; Figure 6B), suggesting that nodes exhibiting higher dynamic modular variability over time tend to have greater amyloid deposits in AD.

**Figure 7.**
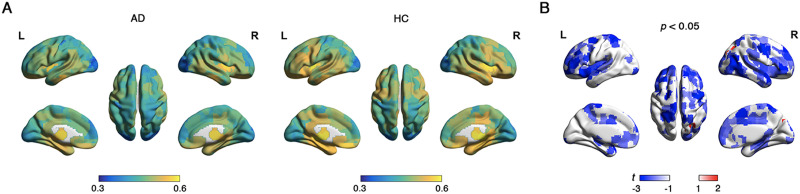
The nodal temporal variability. (A) Spatial distribution of nodal temporal variability in the AD and HC groups. The color bar represents the values of nodal temporal variability, with higher values indicating greater fluctuations in node activity over time. (B) Between-group differences based on the *t* test. Red coloring indicates higher *t* values in AD compared with HC, whereas blue coloring indicates lower *t* values in AD relative to HC.

As nodal temporal variability increases, nodal functional connectivity changes more frequently between windows. The distribution of nodal temporal variability in the AD and HC groups was visually similar (Figure 7A). Specifically, most significant between-group differences in the nodal temporal variability revealed that AD had lower nodal temporal variability than HC (*p* < 0.05, 1.980 < |*t*| < 3.498, 0.335 < |*d*| < 0.628, non-FDR corrected; Figure 7B), except for one node in the right superior occipital cortex. Notably, aberrant changes of dynamic indicators in both modular variability and nodal temporal variability were discovered in the frontal and inferior temporal cortex in AD. Finally, we identified significant positive correlations between modular variability and nodal temporal variability (AD: *r* = 0.810, *p* < 0.001; HC: *r* = 0.825, *p* < 0.001, Supporting Information Figure S2). This observation indicates a close correlation between the variability of node connectivity attributes and the variability in module assignment. For the validation analysis, the findings resembled those observed in the main analyses (Supporting Information Figures S3–S7).

### Relationship With Cognitive Performance

**Figure 8.**
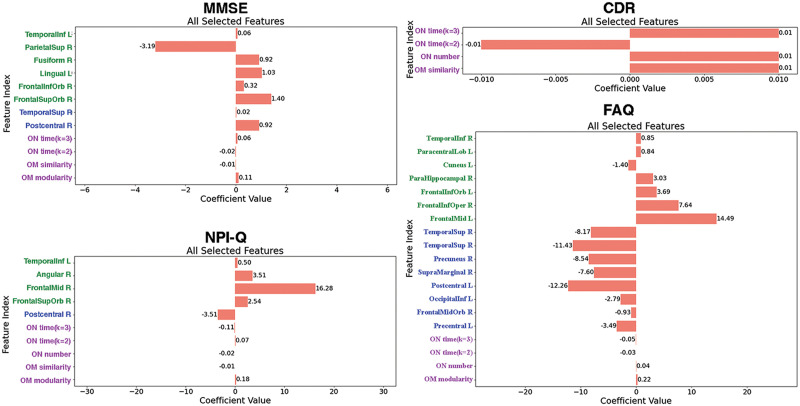
All brain characteristics selected by Lasso regression for each cognitive performance measure. Features are color-coded: green labels represent dynamic nodal temporal variability, purple labels indicate characteristics in overlapping models (OMs) and overlapping nodes (ONs), and blue labels correspond to modular variability. Each color bar denotes the weight of the feature, with numerical values labeled next to the bars. The assessments included the CDR, MMSE, NPI-Q, Geriatric Depression Scale (GDS), and FAQ. The following abbreviations correspond to the brain regions depicted in the figure, with “L” denoting the left hemisphere and “R” denoting the right hemisphere: Temporal Inf (Inferior Temporal Gyrus), Parietal Sup (Superior Parietal Lobule), Fusiform (Fusiform Gyrus), Lingual (Lingual Gyrus), Frontal Inf Orb (Inferior Frontal Gyrus, Orbital part), Frontal Sup Orb (Superior Frontal Gyrus, Orbital part), Temporal Sup (Superior Temporal Gyrus), Postcentral (Postcentral Gyrus), Paracentral Lob (Paracentral Lobule), ParaHippocampal (Parahippocampal Gyrus), Frontal Inf Oper (Inferior Frontal Gyrus, Opercular part), Frontal Mid (Middle Frontal Gyrus), Precuneus (Precuneus), SupraMarginal (Supramarginal Gyrus), Occipital Inf (Inferior Occipital Gyrus), Frontal Mid Orb (Middle Frontal Gyrus, Orbital part), Precentral (Precentral Gyrus), Angular (Angular Gyrus), and Frontal Sup Orb (Superior Frontal Gyrus, Orbital part).

Using Lasso regression, we conducted an analysis to examine the relationships between cognitive performance and brain characteristics (Figure 8). The results revealed that Mini-Mental State Examination (MMSE) scores were associated with overlapping modularity, modular variability in the right postcentral gyrus, and nodal temporal variability in the right superior parietal cortex, right orbitosuperior frontal cortex, left lingual gyrus, right fusiform gyrus, and left inferior temporal cortex (mean squared error [MSE] = 7.354, R-squared [*R*^2^] = 0.256). For the Neuropsychiatric Inventory Questionnaire (NPI-Q), significant associations were identified with overlapping modularity, overlapping modular similarity, modular variability in the right postcentral gyrus, and nodal temporal variability in the right middle frontal cortex, right orbitosuperior frontal cortex, and right angular gyrus (MSE = 1.677, *R*^2^ = 0.886). Regarding the Clinical Dementia Rating (CDR), relevant factors included overlapping modular similarity, the number of overlapping nodes, and membership diversity of overlapping nodes (*k* = 2, 3) (MSE = 0.072, *R*^2^ = 0.147). Finally, Functional Activities Questionnaire (FAQ) scores were primarily associated with overlapping modularity, modular variability in the left postcentral gyrus, right superior temporal cortex, and right precuneus, as well as nodal temporal variability in the left middle frontal cortex (MSE = 5.535, *R*^2^ = 0.875). These findings contribute to a more nuanced understanding of the intricate relationships between cognitive performance and diverse neuroanatomical characteristics.

## DISCUSSION

The functional network of brain tends to organize into overlapping modules (Gu et al., 2022; Lin et al., 2018; Yeo et al., 2014), which are dynamic and continually change their connectivity patterns over time (Braun et al., 2015; Finc et al., 2020; Liu et al., 2020). This study examined these aspects to elucidate alterations associated with AD. Compared with HC, AD exhibited increased overlapping modules and decreased overlapping modularity, with altered nodal overlapping probability, particularly in the superior frontal cortex and hippocampus. Higher nodal overlapping probability was consistently correlated with greater functional flexibility. Additionally, AD exhibited larger dynamic modularity and reduced temporal modular variability in the frontal cortex. Furthermore, increased temporal modular variability and higher nodal overlapping probability were correlated with greater amyloid deposits in AD. Correlations between modular overlapping percentage, overlapping module variability, nodal temporal variability, and cognitive performance (e.g., MMSE) suggest potential biomarkers and therapeutic targets for preserving cognitive function in AD.

### Abnormal Changes of Overlapping Module in AD

Modularity quantifies a network’s ability to decompose into sparsely interconnected, densely intraconnected modules (Newman & Girvan, 2004), which can change with environmental circumstances such as the onset of AD. The increase in the number of overlapping modules among AD likely reflects a decrease in overlapping modularity, indicating altered brain network organization. Such alterations align with “network fragmentation,” where the network divides into inadequately connected modules due to reduced interregional connectivity or hub node disruption (Fornito et al., 2015). In AD, fragmentation of specific modules (e.g., insular) (de Haan et al., 2012) to the increased number of overlapping modules. Moreover, decreased modularity is also evident in nonoverlapping functional modules in the early stages of AD (Brier et al., 2014). Overlapping modularity may serve as a more sensitive indicator than nonoverlapping modularity in AD detection.

A notable decrease was observed in the similarity of individual overlapping modular structures in AD, reflecting significant interindividual variability. This finding aligns with previous studies reporting substantial individual differences in brain function (Verdi et al., 2023) and cognitive/behavioral performance in AD (Tromp et al., 2015). During AD progression, distinct patient subgroups demonstrate varying physiological markers, such as patterns of brain atrophy (Dubois et al., 2016). The reduced similarity in overlapping modular structures may contribute to increased divergence in brain structure and cognitive/behavioral function (Gu et al., 2022). This finding underscores the importance of personalized precision medicine approaches in AD (Toups et al., 2022).

In AD patients, the observed decrease in nodal overlapping probability compared with HC may be attributed to abnormal functional connectivity, which is influenced by the characteristic pathological changes associated with the disease. Related regions were mostly located in the default mode network (DMN), which is a key target of AD neurodegeneration (Dai et al., 2015; Perovnik et al., 2023). Among these regions, the hippocampus, which is pivotal for learning and memory, undergoes early-stage impairment in AD, marked by rapid tissue loss and functional disconnection from other brain regions (Rao et al., 2022). In contrast, regions in the DMN with increased overlapping probability in AD are spatially proximate to those exhibiting decreased probability, indicating a potential compensatory mechanism in response to the challenges posed by AD (Grady et al., 2003; Wiepert et al., 2017).

### Abnormal Changes of Dynamic Module in AD

Dynamic modularity likely captures temporal changes in brain network information processing (Liao et al., 2017). Previous studies have identified that AD patients experience increased transitions between different brain states, particularly those of relatively low intensity, which may be attributed to hypometabolism (Edison et al., 2007; Gu et al., 2020). These transitions may explain the elevated dynamic modularity observed in AD compared with HC. Additionally, increased dynamic modularity in AD aligns with studies linking elevated modularity to low cognitive-control states (Ray et al., 2020). The increased dynamic modularity in AD might also suggest a relationship between the AD-affected brain and reduced cognitive performance. Recent findings support that the human brain undergoes network failure as AD progresses (Ye et al., 2022). Progressive network segregation in AD may disrupt information flow between regions, likely contributing to clinically observed cognitive impairment. Longitudinal studies of dynamic modularity changes could elucidate relationships between these shifts, cognitive decline, and symptom progression, potentially informing therapies to target network dysfunction and slow AD progression. Furthermore, our results indicate increased segregation in AD dynamic multilayer networks, contrasted by reduced static modular overlap, suggesting diminished organizational integration. Temporal modular variability, most pronounced in the frontal and temporal cortices—regions central to AD pathology (Dai et al., 2015, 2019; Perovnik et al., 2023; Verdi et al., 2023)—reinforces disrupted network organization. These findings warrant further investigation to clarify their mechanistic relationships to AD pathology.

Our analysis revealed reduced nodal temporal variability in AD, reflecting diminished functional connectivity adaptability over time. This deficit was most pronounced in AD-vulnerable frontal and inferior temporal cortices (Dai et al., 2019; Verdi et al., 2023), suggesting that nodal temporal variability may serve as a potential biomarker for AD detection (Gu et al., 2020).

Additionally, nodal temporal variability was positively correlated with modular variability. This finding suggests that nodes with stable connectivity maintained consistent modular assignments, while temporally variable nodes frequently shifted their affiliations. This interaction likely influences the brain’s modular organization and could affect cognitive functions, particularly in the context of AD. These findings underscore the disrupted dynamics of modular networks in AD, offering insights into neurodegeneration mechanisms and clinical symptom emergence.

### Characteristics of Overlapping Nodes in AD

Overlapping nodes play a critical role in integrating information across different functional systems. Regions with higher nodal overlapping probability tend to exhibit greater functional flexibility in both groups, which differs from patterns observed in other disorders, such as schizophrenia (Hu et al., 2025). Previous studies have suggested that flexible regions are associated with a broader range of cognitive components and participate in more cognitive tasks (Gu et al., 2022; Lin et al., 2018; Yeo et al., 2015). Our findings suggest that overlapping nodes may underlie cognitive impairment in AD, as flexible regions within overlapping modules may be behaviorally relevant.

Nodes exhibiting higher overlapping probabilities tend to display more dynamic changes over time. This suggests that in AD, brain regions with a greater tendency to overlap in their functional roles and connectivity patterns also demonstrate increased variability in their modular organization over time. In AD patients, both increased temporal modular variability and higher nodal overlapping probability have been correlated with greater amyloid deposition. This variability and overlapping probability are associated with the presence of amyloid plaques, a hallmark of AD pathology (Ma et al., 2022). The abnormal changes observed in overlapping nodes may reflect the brain’s attempt to compensate for the disruptions caused by amyloid deposition in AD.

These results highlight the critical role of overlapping nodes in the pathophysiology of AD. These overlapping nodes may represent areas where neurodegenerative processes are particularly pronounced, underscoring their potential as key indicators of disease progression (Edison et al., 2007; Ma et al., 2022). Future research may elucidate the functional significance of these patterns and their potential diagnostic and therapeutic implications.

### Modular Characteristics Correlation With Cognitive Performances

In this study, the intergroup differences identified, such as overlapping modularity, overlapping modular similarity, dynamic modular variability, and nodal temporal variability, were highly correlated with MMSE, CDR, NPI-Q, and FAQ scores, underscoring the potential influence of network organization on cognitive function. The most critical regions, which demonstrate high potential as biomarkers, were located in the inferior frontal and temporal cortices and the postcentral gyrus. These regions have been previously associated with early AD prediction (Adaszewski et al., 2013). The strong correlations between these modular organization-related characteristics and behavioral indicators likely provide profound insights into cognitive dysfunction, particularly in the context of early diagnosis and the development of treatment strategies.

### Limitation

Considering the possibility of mislabeling due to clinical phenotype and pathology mismatch (Snowden et al., 2011), our study focuses on the correlation between module-related measures and SUVR at the regional level. However, future research should integrate more disease-specific biomarkers, such as tau and cerebrovascular markers, to better investigate disease heterogeneity. In addition, studying the variations in overlapping modules within mild cognitive impairment may contribute to a better understanding of AD progression. It would also be beneficial to use individualized parcellations to derive and compare brain network topology (Chong et al., 2017). Furthermore, we did not compare different sliding window lengths and steps, which may affect the dynamic multilayer modular structure. Future research could explore the impact of varying sliding window parameters on dynamic modular structure.

### Conclusions

This study demonstrates that AD disrupts both static overlapping modular architecture and dynamic network reconfiguration, characterized by increased overlapping modules, abnormal nodal overlapping probability, and decreased nodal temporal variability, particularly in AD-vulnerable frontal and temporal regions. These network disruptions were strongly correlated with cognitive/behavioral assessments, underscoring their clinical relevance as potential biomarkers for disease progression. The findings suggest that overlapping nodes play a critical role in AD pathophysiology, serving as important components of overlapping modularity that relate to network flexibility and potentially reflecting compensatory mechanisms in response to amyloid deposition. These insights may advance the understanding of neural degradation mechanisms and highlight the importance of overlapping node analysis in developing targeted therapeutic interventions for AD.

## METHODS

### Participants

All resting-state fMRI data were acquired from the Alzheimer’s Disease Neuroimaging Initiative (ADNI) dataset (ADNI-2/3 phases, https://adni.loni.usc.edu/) using standardized scanning parameters. The diagnostic criteria are detailed in the ADNI manual (https://www.adni-info.org), which specifies a CDR score of ≥0.5, an MMSE score of <26, and adherence to the National Institute of Neurological and Communicative Disorders and the Stroke/Alzheimer’s Disease and Related Disorders Association criteria for probable AD. Participants who met these criteria were assigned to the AD group in the current study. All participants completed neuropsychological assessments including the NPI-Q and FAQ. HCs met the following inclusion criteria: (a) absence of neurological or psychiatric disorders, (b) no abnormal findings such as infarction or focal lesions on conventional brain MRI, and (c) MMSE score ≥26. After applying rigorous quality control measures to exclude data with image artifacts, incomplete scans, and excessive head motion during preprocessing, the final sample comprised 64 AD patients and 61 age-matched HCs. Demographic and clinical characteristics of the participants are presented in Table 1.

**Table 1.** Demographic and clinical variables.

	AD	HC	*p*
Age	74.04 (8.28)	74.86 (7.25)	0.558
Gender	30 M, 34 F	30 M, 31 F	0.859
MMSE	21.58 (3.17)	29.26 (0.89)	<0.001*
CDR	0.5 (*n* = 21), 1(*n* = 33), 2 (*n* = 1)	0 (*n* = 55), 0.5 (*n* = 2)	<0.001*
NPI-Q	4.59 (3.92)	0.47 (1.05)	<0.001*
GDS	1.61 (1.56)	0.86 (1.04)	0.267
FAQ	17.83 (6.79)	0.05 (0.22)	<0.001*

*Note*: Data are presented as means (standard deviations, *SD*). The two-sample two-tailed *t* test was performed to examine between-group differences in age and MMSE, Mann-Whitney *U* test was used for NPI-Q, GDS, and FAQ, and chi-square test was performed for gender and CDR. The asterisk indicates a significant between-group difference (*p* < 0.05). There was nine AD and four HC who lacked CDR data. Abbreviation: AD, Alzheimer’s disease; CDR, Clinical Dementia Rating; HC, healthy control; MMSE, Mini-Mental State Examination; NPI-Q, Neuropsychiatric Inventory Questionnaire; GDS, Geriatric Depression Scale; FAQ, Functional Activities Questionnaire.

### Data Acquisition and Preprocessing

The MRI images of each subject were obtained with 3.0 T scanners. MRI acquisitions were performed according to the ADNI 2 and 3 acquisition protocol (Jack et al., 2008). Resting state-fMRI was obtained using an echo-planar imaging sequence and the following parameters. Within the dataset, our analysis exclusively incorporates data with a repetition time (TR) of 3,000 ms and an echo time of 30 ms. Image preprocessing for R-fMRI was carried out using the Data Processing Assistant for Resting-State fMRI (https://rfmri.org/DPARSF) (Yan & Zang, 2010) toolbox and SPM8 (https://www.fil.ion.ucl.ac.uk/spm). For each participant, the first five scans were discarded before preprocessing. The realignment was performed after slice timing to the first volume to correct head motion. Four AD were excluded for excess head motion (>3 mm/3°). Then, the T1-weighted image was coregistered to the mean functional image after motion correction and then segmented into gray matter, white matter, and cerebrospinal fluid tissue images. The head motion-corrected functional images were further spatially normalized to the Montreal Neurological Institute space using the parameters estimated from T1 unified segmentation (Ashburner & Friston, 2005) and were resampled into 3-mm isotropic voxels. Finally, to reduce the influence of artifacts, the normalized functional images were detrended, regressed out the nuisance variables (Friston’s 24 head motion parameters, global signals, white matter, and cerebrospinal fluid signals), and bandpass filtered (0.01–0.08 Hz).

### Construction of Brain Static and Dynamic Functional Networks

The brain functional network construction was carried out using the graph theoretical network analysis (GRETNA, https://www.nitrc.org/projects/gretna/) (Wang et al., 2015). Network nodes were defined as 625 similarly sized brain regions based on a refined automated anatomical labeling atlas (Crossley et al., 2013; Dai et al., 2019; Tzourio-Mazoyer et al., 2002; Zalesky et al., 2010). For static functional network construction, Pearson correlation coefficients were computed between time series of all node pairs, generating symmetric correlation matrices. Given the ambiguous biological interpretation of negative correlations (Murphy et al., 2009), only positive correlations were retained while negative values were set to zero. Binary undirected functional networks were then created by applying a 15% sparsity threshold. Setting the sparsity at 0.15 strikes a balance between sensitivity (detecting genuine connections) and specificity (avoiding false positives) (Bordier et al., 2017; Gu et al., 2022; Lennartz et al., 2018; Mohammadian et al., 2023; Wee et al., 2012). Dynamic functional networks were generated using a sliding window approach with a window length of 50 TRs and a sliding step of two TRs. The 50-TR window length has been widely used in previous studies, for example, those that apply dynamic sparse connectivity models (Allen et al., 2014; Rubinov & Sporns, 2010). The step size determines the temporal resolution of the dynamic connectivity analysis. A smaller step size, such as our choice of two TRs, allows for a more finely sampled representation of the changes in functional connectivity over time (Li et al., 2024).

For each participant, this yielded binary undirected dynamic networks at 15% sparsity, resulting in 43 temporal networks for ADNI-2 participants and 72 temporal networks for ADNI-3 participants. A validation analysis using equal numbers of matrices from each dataset is presented in the Supporting Information.

### Detection of Overlapping Modules for Static Functional Networks

Individual overlapping modules on static functional networks were detected using the MCMOEA. This algorithm does not require a priori specification of the number of overlapping modules and instead evolves a population of candidate module structures using customized operators to achieve optimal balance between maximizing intramodule link density and minimizing intermodule link density. The MCMOEA has demonstrated superior performance compared with various state-of-the-art algorithms on both synthetic and real-world networks (Wen et al., 2017). The implementation procedure followed our previous studies on brain functional overlapping module detection in adults (Gu et al., 2022; Lin et al., 2018), with detailed parameters provided in Supporting Information Section 1.1.

### Analysis of Static Overlapping Module Characteristics

Eight measures were used to capture the characteristics of overlapping modules and overlapping nodes (Gu et al., 2022). For overlapping modules, three characteristics were calculated for each participant: (a) the number of overlapping modules; (b) the overlapping modularity score (*Mod*^*ov*^), which indicates the quality of module separation in a network (Lázár et al., 2010; Supporting Information Section 1.2); and (c) the modular similarity, obtained by calculating the average generalized normalized mutual information score between each participant’s overlapping modular structure and specified counterparts.

The overlapping nodes, which participate in two or more functional modules, play essential roles in promoting network communication and functional flexibility (Gu et al., 2022; Lin et al., 2018; Yeo et al., 2014). We first characterized the spatial patterns of overlapping nodes by visualizing the distribution of nodal overlapping probability, estimated as the percentage of participants in which each corresponding brain region was an overlapping node. Four additional characteristics of overlapping nodes were then calculated for each participant: (a) the number of overlapping nodes; (b) the membership diversity of overlapping nodes, obtained by counting nodes participating in *k* modules (*k* ≥ 2); (c) the modular overlapping percentage regarding the seven classic nonoverlapping functional modules (Yeo et al., 2011), calculated by dividing the number of overlapping nodes in each module by the total number of overlapping nodes; and (d) the spatial variability of overlapping nodes, calculated as the average Jaccard distance between overlapping node sets from each participant and specified counterparts.

### Tracking Dynamic Functional Modular Structures

Dynamic functional connectivity offers greater insight into plasticity and dynamics of brain network (Finc et al., 2020). It captures spontaneously recurring connectivity patterns, enabling observation of their temporal evolution to uncover mechanistic insights into brain function and cognition. This capability is critical for probing temporal variability in intrinsic modular organization. To investigate overlapping modular architecture, we analyzed functional modules using dynamic multilayer network modeling.

The multilayer network model identifies time-varying modular structures by linking networks across consecutive time windows, which provides important statistical benefits for network module estimation (Liu et al., 2020). Using this model, we investigated the community structure of multi-window networks. This framework integrates individual networks via interwindow nodal links, enforcing temporal continuity of modular architecture. The optimal modular structure was identified by maximizing the multilayer modularity index *Mod*_*multi*_, which quantifies the extent of segregation between network modules:

$${\text{Mod}}_{\text{multi}}\left(\gamma ,\omega \right)=\frac{1}{2\mu }\sum_{\text{ijsr}}\left[\left(A_{ijs}-\gamma_{s}\frac{k_{\text{is}}k_{\text{js}}}{2m_{s}}\right)\delta \left(s,r\right)+\delta \left(i,j\right)\omega_{jsr}\right]\delta \left(M_{\text{is}},M_{\text{jr}}\right),$$

where *i* and *j* are node labels, *s* and *r* are window labels, and *μ* denotes the total degree of the multilayer network. For window *s*, the *A*_*ijs*_ denotes the connectivity strength between nodes *i* and *j*, *k*_*is*_ denotes the degree of node *i*, and *m*_*s*_ denotes the total degree. The function *δ*(*x*, *y*) equals 1 if *x* equals *y*, and equals 0 otherwise. The topological resolution parameter *γ*_*s*_ determines the detected module size in window *s*. The temporal coupling parameter *ω*_*jsr*_ denotes the strength of interlayer coupling for node *j* between window *r* and *s*. As usual, we set the default values ***γ*** and ***ω*** to 1.

To assess the time-varying nature of modular architecture, we calculated modular variability for each node to quantify how frequently individual nodes dynamically switch their module affiliation over time (Liao et al., 2017; Supporting Information Section 1.3). Higher modular variability indicates more frequent switching between modules. Given the heuristic nature of the multilayer Louvain algorithm (Blondel et al., 2008) and near-degeneracy of the modularity landscape, partition solutions can vary across algorithm runs. To address this degeneracy, we repeated multilayer modular detection 100 times for each subject and averaged module-relevant measures (modularity and modular variability) across all repetitions.

Moreover, for a more comprehensive understanding of individual node contributions to their respective modules, we further investigated the temporal variability (*V*_*k*_) of functional connectivity for each node (Gu et al., 2020; Zhang et al., 2016; Supporting Information Section 1.4).

### Statistical Analysis

The permutation test (*N* = 10,000) was performed to quantify the between-group differences in above overlapping module-related characteristics, which randomly shuffled group labels to build null models. To explore the functional significance of nodal overlapping and variability measures, we computed Pearson correlation coefficients among group-level nodal measures (nodal overlapping probabilities, dynamic modular variability, and temporal variability). Functional flexibility, defined as the capacity of brain regions to participate in multiple cognitive processes and adaptively shift their functional roles based on varying demands, represents a fundamental property of neural networks that enables efficient information integration across functional domains. To assess this property, we examined correlations between our nodal characteristics and empirically derived functional flexibility data from 10,449 cognitive experiments (Yeo et al., 2015). Higher functional flexibility values indicated greater probability of regional activation across diverse cognitive components, reflecting enhanced versatility in supporting multiple functional processes. Additionally, the SUVR serves as a critical metric for assessing amyloid deposition in the brain in AD. Positron Emission Tomography scans were conducted using Pittsburgh InBound to visualize amyloid deposits in the brains of 32 cognitively impaired patients with AD (La Joie et al., 2020). Individual SUVR maps were averaged to create a composite map, allowing us to investigate whether overlapping nodes are associated with amyloid deposition. For AD patients, the correlation between nodal characteristics and SUVR were calculated. The FDR correction was applied with the threshold *p* < 0.05. All results presented were FDR-corrected unless stated otherwise. Finally, Lasso regression analysis (default alpha = 0.1) was performed to examine associations between brain network characteristics and cognitive performance in AD patients.

## ACKNOWLEDGMENTS

This work was supported by the STI2030-Major Projects (No. 2022ZD0213300), the National Natural·Science Foundation of China (No. 32371147), the Guangdong Basic and Applied Basic Research Foundation (No. 2022A1515012005).

Data collection and sharing for Alzheimer’s Disease Neuroimaging Initiative (ADNI) was funded by National Institutes of Health Grant U01 AG024904 and the Department of Defense (Award Number W81XWH-12-2-0012). ADNI is funded by the National Institute on Aging, the National Institute of Biomedical Imaging and Bioengineering, and through generous contributions from the following: AbbVie, Alzheimer’s Association; Alzheimer’s Drug Discovery Foundation; Araclon Biotech; BioClinica, Inc.; Biogen; Bristol-Myers Squibb Company; CereSpir, Inc.; Cogstate; Eisai Inc.; Elan Pharmaceuticals, Inc.; Eli Lilly and Company; EuroImmun; F. Hoffmann-La Roche Ltd. and its affiliated company Genentech, Inc.; Fujirebio; GE Healthcare; IXICO Ltd.; Janssen Alzheimer Immunotherapy Research & Development, LLC.; Johnson & Johnson Pharmaceutical Research & Development LLC.; Lumosity; Lundbeck; Merck & Co., Inc.; Meso Scale Diagnostics, LLC.; NeuroRx Research; Neurotrack Technologies; Novartis Pharmaceuticals Corporation; Pfizer Inc.; Piramal Imaging; Servier; Takeda Pharmaceutical Company; and Transition Therapeutics. The Canadian Institutes of Health Research is providing funds to support ADNI clinical sites in Canada. Private sector contributions are facilitated by the Foundation for the National Institutes of Health (www.fnih.org). The grantee organization is the Northern California Institute for Research and Education, and the study is coordinated by the Alzheimer’s Therapeutic Research Institute at the University of Southern California. ADNI data are disseminated by the Laboratory for Neuro Imaging at the University of Southern California.

## SUPPORTING INFORMATION

Supporting information for this article is available at https://doi.org/10.1162/NETN.a.516.

## AUTHOR CONTRIBUTIONS

Yue Gu: Data curation; Formal analysis; Investigation; Methodology; Project administration; Resources; Software; Supervision; Validation; Visualization; Writing – original draft; Writing – review & editing. Ying Lin: Conceptualization; Investigation; Methodology; Resources; Software; Writing – review & editing. Liangfang Li: Formal analysis; Validation; Visualization; Writing – review & editing. Junji Ma: Methodology; Software; Visualization; Writing – review & editing. Sihan Wei: Software; Visualization; Writing – review & editing. Zhengjia Dai: Conceptualization; Funding acquisition; Investigation; Methodology; Project administration; Resources; Supervision; Writing – review & editing.

## ETHICS STATEMENT

We confirm that we have read the Journal’s position on issues involved in ethical publication and affirm that this report is consistent with those guidelines.

## FUNDING INFORMATION

Zhengjia Dai, STI2030-Major Projects, Award ID: 2022ZD0213300. Zhengjia Dai, National Natural·Science Foundation of China, Award ID: 32371147. Zhengjia Dai, Guangdong Basic and Applied Basic Research Foundation, Award ID: 2022A1515012005.

## DATA AVAILABILITY STATEMENT

The original data that support the findings of this study are available from the ADNI dataset.

## Supplementary Material



## TECHNICAL TERMS

fMRI (functional magnetic resonance imaging): A neuroimaging technique that measures brain activity by detecting changes in blood flow.
Resting-state fMRI: A technique used to assess brain activity when a subject is not performing any specific task.
Overlapping node: A single brain region involved in multiple functional modules.
Overlapping module: Groups of brain regions that share connections, participating in multiple cognitive processes simultaneously.
Dynamic multilayer modules: Structures representing varying brain connectivity patterns across different layers or modalities over time, reflecting complex interactions.
Temporal variability: Fluctuations in brain activity patterns over time, indicating changes in functional connectivity among different time windows.
Sliding window: A method for analyzing time-varying data by using a moving time frame to capture dynamic changes in connectivity.
