# Supplementary material for: Spatiotemporal profiling of functional network overlapping modules in Alzheimer’s disease
Source: Netw Neurosci. 2026 Jan 28;10(1):185–203. doi: 10.1162/NETN.a.516 (PMC12956295; doi:10.1162/NETN.a.516)
Supplement: Supplementary file 1 [file netn-10-1-185-s001.pdf]

Supplementary

1 Methods

1.1 The Application Procedure of MCMOEA

We used the maximal clique-based multiobjective evolutionary algorithm (MCMOEA) to detect individual overlapping modules on static functional networks. Initially, a population of 100 candidate overlapping modular structures was generated. Each was linked to a single-objective optimization problem and was obtained using the Tchebycheff method to decompose the original multi-objective optimization problem(Mietinnen, 1999). Here, the MCMOEA evolved the population until the maximum number of generations reached 10,000, or the population stopped updating for 500 consecutive generations. As a result, the last generation of the population was returned. It contained 100 non-dominated overlapping modular structures with a near-optimal trade-off between intra- and inter-link densities. Due to its probabilistic nature, we executed MCMOEA for 100 times. Following that, the fast nondominance ranking method(Deb et al., 2002) was used to find nondominated solutions among the resulting 10,000 solutions. After the removal of duplicate solutions, the similarity between two different non-dominated solutions was calculated by generalized normalized mutual information (gNMI)(Lancichinetti et al., 2008). The gNMI is an extension of mutual information in the context of overlap and has been shown to be reliable(Danon et al., 2005). Higher gNMI values imply that two overlapping module structures are more similar to each other. As such, the solution with the largest average gNMI was considered to reveal the most typical and robust overlapping module structure for each participant and was selected for subsequent analysis.

1.2 Analyses Characteristics of Static Overlapping Modules

The overlapping modularity score (Lázár et al., 2009) Mod^{ov} is calculated,

$$Mod^{ov} = \frac{1}{N}\sum_{r=1}^{N}\left[\frac{\sum_{i\in OM_r}\frac{\sum_{j\in OM_r, i\neq j} a_{i,j} - \sum_{j\notin OM_r} a_{i,j}}{d_i \times s_i}}{n_r} \times \frac{n_r^e}{\binom{n_r}{2}}\right]$$

where $a_{i,j}$ denotes an element in the adjacency matrix of the network ($a_{i,j} = 1$ for nodes *i* and *j* being connected, $a_{i,j} = 0$ for the opposite), and d_i denotes the degree of node *i*. The s_i is the number of overlapping modules node *i* belongs to, n_r and n_r^e are the numbers of nodes and edges in OM_r, respectively, and *N* is the number of overlapping modules. Since the first factor $\frac{\sum_{j\in OM_r, i\neq j} a_{i,j} - \sum_{j\notin OM_r} a_{i,j}}{d_i \times s_i}$ ranges between -1 and 1, the second factor $\frac{n_r^e}{\binom{n_r}{2}}$ between 0 and 1, the whole expression varies between -1 and 1. When the Mod^{ov} value approaches 1, it indicates better separation of modules, while approaching -1 suggests weaker separation.

1.3 The Dynamic Modular Variability

For modular variability of a given node *k* between two modular structures in window *s* and *r*,

$$MV_k(s,r) = 1 - \frac{|X_k(s) \cap X_k(r)|}{|X_k(s)|} \cdot \frac{|X_k(s) \cap X_k(r)|}{|X_k(r)|}$$

where $X_k(s)$ and $X_k(r)$ denote the module to which node *k* belongs in window *s* and *r*, respectively. $|X_k(s) \cap X_k(r)|$ denotes the number of common nodes in the two modules. A small overlap between the two modules $X_k(s)$ and $X_k(r)$ indicates large module affiliation variability. Then, the total modular variability across all of the *n* windows can be evaluated as

$$MV_k = \sum_{s=1}^{n} w_s MV_k(s)$$

where $MV_k(s) = \sum_{s \neq r} MV_k(s, r) / (n - 1)$ denotes the modular variability for node *k* between module structure in window *s* and all other windows. A normalized weighed coefficient w_s was used to reduce the bias of potential outlier time windows. The w_s denotes the spatial similarity in the modular architecture between window *s* and all other windows, and is estimated using adjusted mutual information(Vinh et al., 2010).

1.4 The Nodal Temporal Variability

To calculate the perspective of temporal variability of functional connectivity in each node(Gu et al., 2020; Zhang et al., 2016). For each participant, the nodal temporal variability V_k of node *k* can be described by

$$V_k = 1 - \frac{\sum_{i \neq j} \rho_{F_{i,k}, F_{j,k}}}{n \times (n-1)},$$

where *n* is the total number of windows and $\rho_{F_{i,k}, F_{j,k}}$ is the Pearson correlation coefficient between the functional connectivity profiles of region *k* from *i* and the *j* windows (i, j = 1, 2, …, n; $i \neq j$; k = 1, 2, …, 625).

1.5 Validation Analysis

The dynamic matrices number is 43 for individuals in ADNI-2 and 72 for individuals in ADNI-3, due to the different scanning time. To validate our results, we choose the first 43 matrices from ADNI-3 to make it is equal to ADNI-2. By using the same number of dynamic matrices for all subjects, we reanalysed dynamic functional connectivity as Section 4.6 to exam our main results.

2 Results

2.1 Validation Results

We validated the reliability of our main findings regarding the influence of dynamic matrices number on dynamic functional brain network analysis. By using the same number of dynamic matrices for all subjects, we found that AD was associated with larger dynamic modularity compared to HC (p = 0.023, t = 2.303, Cohen's d (d) = 0.410; Fig.S3). Consistently, the large temporal variability in module affiliations across all time windows was located in the frontal and temporal cortex (Fig.S4A). The AD exhibited significantly decrease temporal modular variability in frontal cortex ($p < 0.05$, $-4.005 < t < -2.003$, $-0.710 < d < -0.356$; Fig.S4B). Similarity, the temporal modular variability was similar to the nodal overlapping probability in the static modular structure (AD: r = 0.244, HC: r = 0.151, $p < 0.001$; Fig.S5). Compared with HC, AD had lower nodal temporal variability ($p < 0.05$, $-4.311 < t < -3.293$, $-0.769 < d < -0.588$, FDR-corrected; Fig.S6). Additionally, there were significant positive correlations between modular variability and nodal temporal variability (AD: r = 0.645, $p < 0.001$; HC: r

= 0.445, $p < 0.001$, Fig.S7). Totally, the observations of validation analysis resembled those found in the main analyses.

3 Supplementary Figures and legends

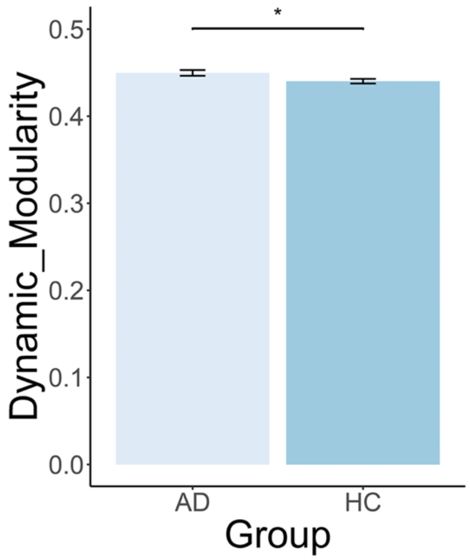

Fig.S1 The differences in dynamic modularity between AD and HC.

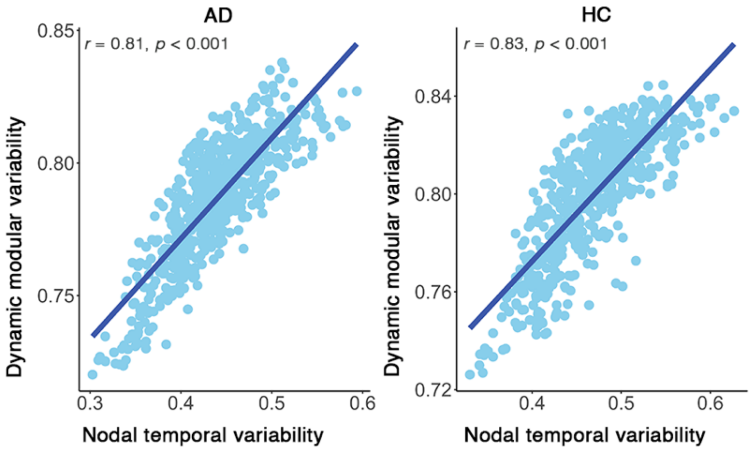

Fig.S2 The correlation between the temporal modular variability and the nodal temporal variability in AD and HC groups. Each point represents a brain node.

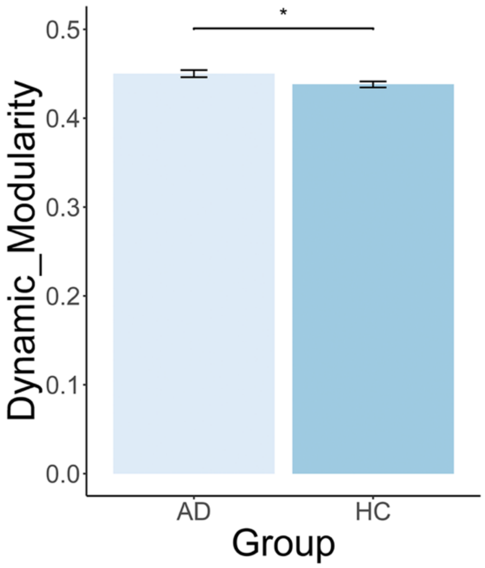

Fig.S3 The differences in dynamic modularity between AD and HC.

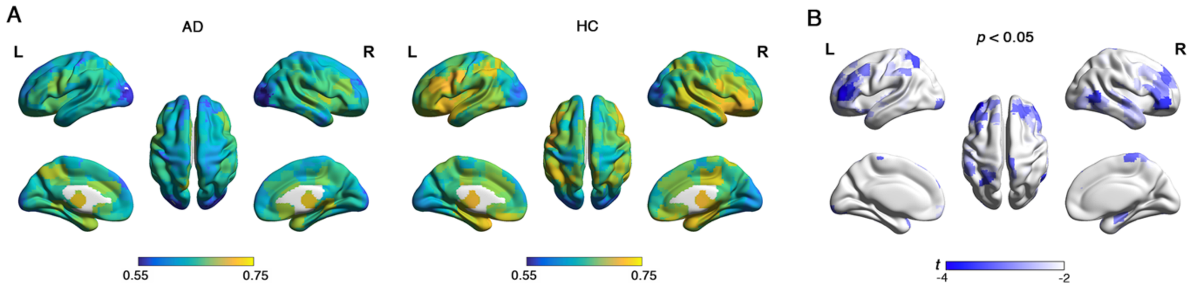

Fig.S4 The temporal variability in module affiliations. (A) Spatial distribution of the temporal variability in module affiliations across all time windows in AD and HC groups. (B) Group comparison showing changes in the temporal variability of AD compared to HC.

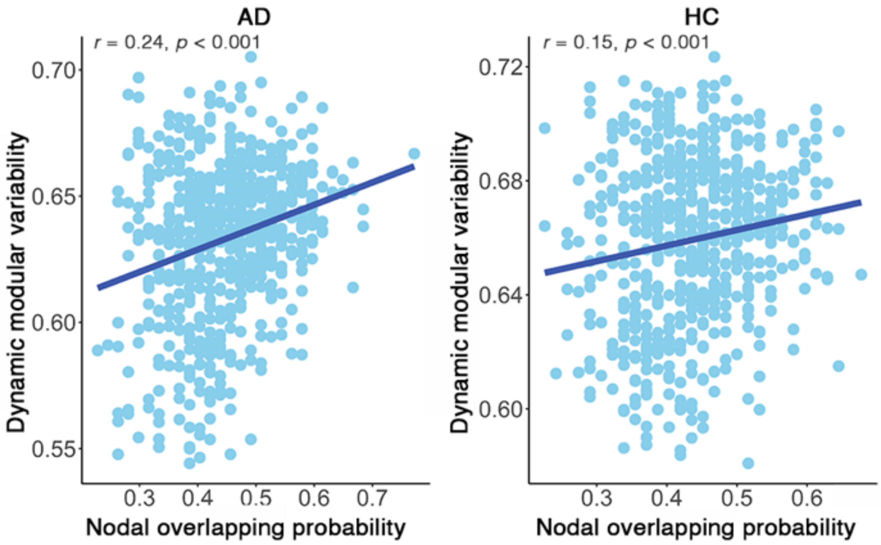

Fig.S5 The correlation between the temporal modular variability and the nodal overlapping probability in AD and HC groups. Each point represents a brain node.

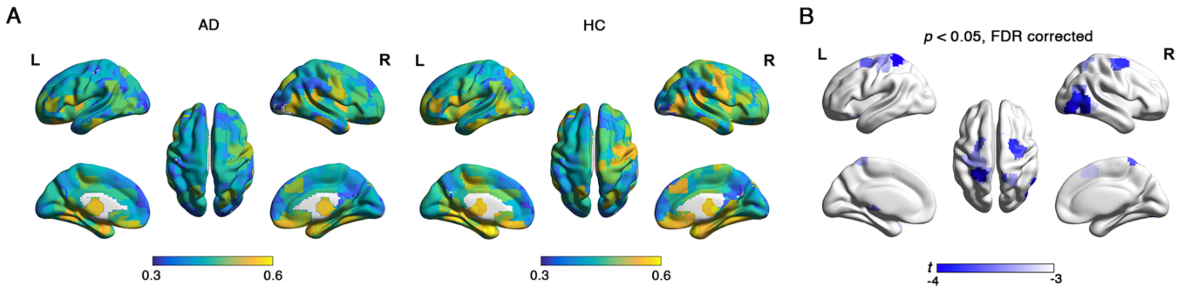

Fig.S6 The nodal temporal variability. (A) The distribution of nodal temporal variability in the AD and HC group, along with (B) between-group differences. The red colour indicates higher values in AD compared to HC, while the blue colour indicates the opposite.

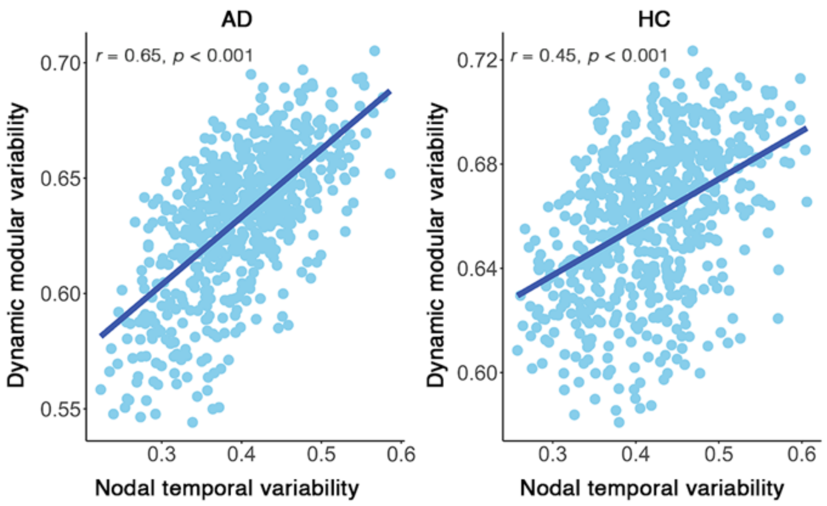

Fig.S7 The correlation between the temporal modular variability and the nodal temporal variability in AD and HC groups. Each point represents a brain node.

References

Danon, L., Diaz-Guilera, A., Duch, J., & Arenas, A. (2005). Comparing community structure identification. *Journal of Statistical Mechanics-Theory and Experiment*, *2005*(09), P09008. https://doi.org/10.1088/1742-5468/2005/09/p09008

Deb, K., Agrawal, S., Pratap, A., & Meyarivan, T. (2002). *A Fast Elitist Non-dominated Sorting Genetic Algorithm for Multi-objective Optimization: NSGA-II*. *6*, 182–197.

Gu, Y., Lin, Y., Huang, L., Ma, J., Zhang, J., Xiao, Y., & Dai, Z. (2020). Abnormal dynamic functional connectivity in Alzheimer's disease. *CNS Neuroscience and Therapeutics*, *26*(9), 962–971. https://doi.org/10.1111/cns.13387

Lancichinetti, A., Fortunato, S., & Kertész, J. (2008). Detecting the overlapping and hierarchical community structure in complex networks. *New Journal of Physics*, *11*(3), 19–44.

Lázár, A., Ábel, D., & Vicsek, T. (2009). *Modularity Measure of Networks With Overlapping Modules*. IOP Publishing, Pages.

Mietinnen, K. M. (1999). International Series in Operations Research and Management Science. In *Non-linear multiobjective optimization*. Kluwer.

Vinh, N. X., Epps, J., & Bailey, J. (2010). Information theoretic measures for clusterings comparison: Variants, properties, normalization and correction for chance. *Journal of Machine Learning Research*, *11*, 2837–2854.

Zhang, J., Cheng, W., Liu, Z., Zhang, K., Lei, X., Yao, Y., Becker, B., Liu, Y., Kendrick, K. M., Lu, G., & Feng, J. (2016). Neural, electrophysiological and anatomical basis of brain- network variability and its characteristic changes in mental disorders. *Brain*, *139*, 2307–2321. https://doi.org/10.1093/aww143
